# Porous Ba Ferrite Prepared from Wood Template

**DOI:** 10.3390/ma2041923

**Published:** 2009-11-20

**Authors:** Nobuyasu Adachi, Masayuki Kuwahara, Chee Kiong Sia, Toshitaka Ota

**Affiliations:** Ceramics Research Laboratory, Nagoya Institute of Technology/ 10-6-29 Asahigaoka, Tajimi, Gifu 507-0071, Japan; E-Mails: kuwahara@crl.nitech.ac.jp (M.K.); sia@crl.nitech.ac.jp (C.K.S.); ota@nitech.ac.jp (T.O.)

**Keywords:** porous ceramics, Ba-ferrite, wood template, magnetic anisotropy

## Abstract

Ba ferrite materials with porous microstructures were prepared from a natural cedar wood template in order to investigate new electromagnetic shielding materials. The wood templates were infiltrated with barium nitrate and iron nitrate solutions (molar ratio = 1:12) and dried to form ferrite gel, then, they were sintered in air at a temperature between 800 °C and 1400 °C. The 1-dimensional porous structures were retained after sintering and the pore size was approximately 10–20 μm. These ferrites show large coercive force and anisotropy field. The largest coercive force was obtained for the specimen sintered at 800 °C.

## 1. Introduction

Porous ceramics have been attractive materials for various applications such as thermal and sound absorption, filtration, catalysts, etc. Many researchers have been studied their properties due to the porous structure [1]. We had developed a unique preparation technique for porous ceramics where the porous structures of the ceramics were copied from a natural wood template by a bio-casting method [2,3,4].

Recently, we have been studying magnetic materials with porous structures for high frequency range electro-magnetic interference (EMI) shielding applications. The GHz electromagnetic noise absorbers become more important as higher frequencies are utilized in wireless and broadband communication systems. Magnetic ferrite ceramics are widely used as electromagnetic absorber materials mainly in the MHz frequency region because of high permeability. Generally, the permeability of bulk samples decreases in the GHz frequency region due to the so-called Snoek’s limit [5]. However, it is reported that thin films of Ni-Zn soft ferrite with a preferred orientation exceed Snoek’s limit and the permeability is kept high in the GHz frequency range [6]. This presents a possibility that the microstructure gives an increase of the permeability of ferrite materials in the GHz frequency region. We already reported the synthesis of a porous Ni-Zn ferrite from a wood template and found different magnetic properties depending on the axis along the 1-dimesional pore structure [7]. We are expecting that thin film-like magnetic properties of the porous ferrite increase the permeability in the high frequency region. We have also been investigating Ba-ferrite with porous structure. The hexagonal Ba-ferrite is a hard magnetic material with large magnetic anisotropy energy and is recently expected to be a new electromagnetic absorber by utilizing magnetic resonance because the resonance frequency with hard Ba-ferrite lies in GHz frequency region due to its large magnetic anisotropy. In this paper, we report the preparation of a porous Ba-ferrite BaFe_12_O_19_ from a wood template by the bio-casting method and its magnetic properties.

## 2. Experimental Procedures

For the wood templates, commercial Japanese cedars were used. The cedars are coniferous trees which have uniform and micro-porous structures. Pieces of raw wood were cut (e.g. 15 mm × 10 mm × 10 mm) and boiled with 25% ammonia for 1 h in order to remove the wood extractive organic compounds, then, they were washed with distilled water and dried in air at 60–80 °C for 1 day.

As starting chemicals, reagent grade Ba(NO_3_)_2_ and Fe(NO_3_)_3_·9H_2_O were weighed to be stoichiometric compositions of BaFe_12_O_19_ and mixed at 60 °C for 10–15 min under stirring. These nitrate solutions were infiltrated into the wood specimens. Finally, the specimens were sintered at 800–1400 °C for 8 h in air. The crystal structures of the specimens were analyzed by X-ray diffractometer (XRD) with Cu K*α* radiation (RINT 1100, Rigaku Co.). The microstructures were observed by scanning electron microscope (SEM; JSM-7100 FX, JEOL Ltd.) and Energy dispersive X-ray spectroscopy (EDX) analysis was also performed. Magnetizations were measured by the vibrating sample magnetometer (VSM; TOEI Co.) 

## 3. Results and Discussion

**Figure 1:**
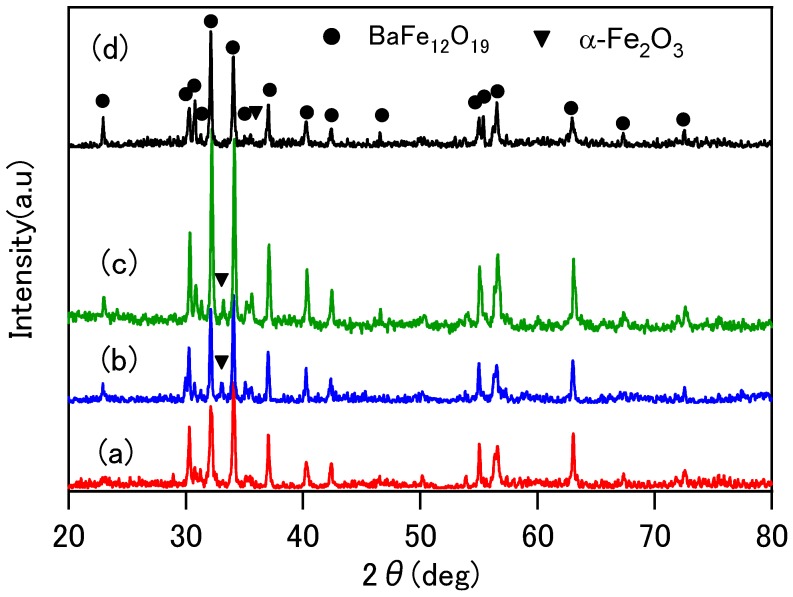
powder XRD patterns of the specimens sintered at: (a) 800 °C, (b) 1000 °C, (c) 1200 °C and (d) 1400 °C for 8 h.

The powder XRD patterns of the sintered specimens are shown in Figure 1. In all specimens, the polycrystalline diffraction peaks from the hexagonal Ba-ferrite BaFe_12_O_19_ phase were observed. The small diffraction peaks from the α-Fe_2_O_3_ phase was also recognized in all specimens.

**Figure 2:**
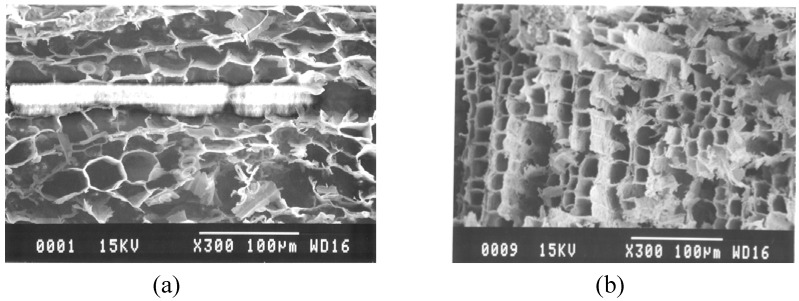
SEM micrographs of the porous Ba-ferrite sintered at: (a) 800 °C and (b) 1000 °C.

The appearance of the specimen sintered at 800 °C retains the grains of the wood template and its color is still brown. The specimens sintered at temperatures of more than 1000 °C become black. From the point of view of strength, the specimens sintered at temperatures below 1000 °C are fragile and difficult to form or cut into another shape. However, the porous structures of the cedar wood are maintained after sintering. The microstructures of the sintered specimens were shown in Figure 2. The pore size was approximately 10–20 μm and thickness of the cell wall is approximately 1–2 μm, which is the typical size reported before [2]. From these images, it can be concluded that the cell wall of the original cedar wood transformed to ferrite ceramics. The EDX analysis indicates that the composition ratio of Ba:Fe is 1:14.3. This result reflects the XRD analysis where the Ba-ferrite BaFe_12_O_19_ phase and α-Fe_2_O_3_ phase co-exist. 

**Figure 3:**
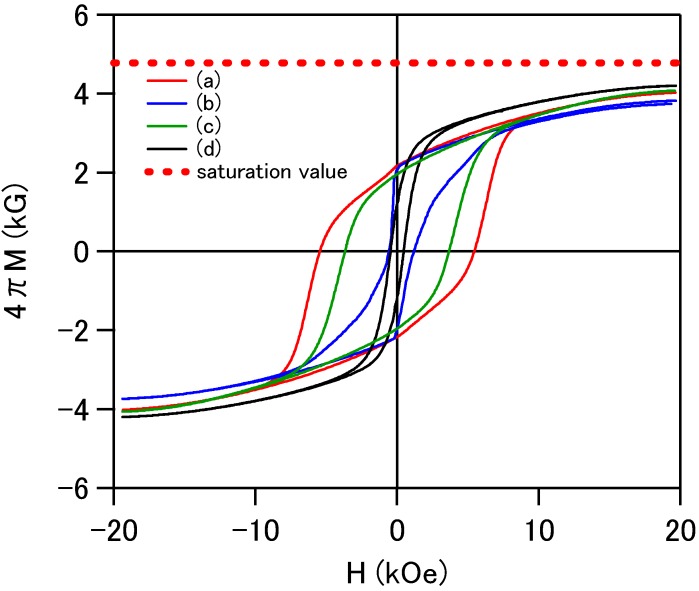
Magnetic hysteresis loops of powder forms of the specimens sintered at: (a) 800 °C, (b) 1000 °C, (c) 1200 °C and (d) 1400 °C.

The magnetic hysteresis curves shown in Figure 3 were measured by using the powder forms of the sintered specimens. The magnetization curves do not saturate even at 20 kOe, which indicates a large anisotropy field of the hexagonal Ba-ferrite materials. As far as the saturation magnetization *M*_s_ = 4.8 kgauss of the BaFe_12_O_19_ is concerned [8], the extrapolated *M*_s_ values of the specimens almost agree with the reference value. The specimen sintered at 800 °C shows the largest coercive force of 8 kOe. With an increase of the sintering temperature, the coercive force tends to decrease. The coercive force is intimately related with grain size because large grain growth often makes a multi-magnetic domain structure. Single magnetic domain structure is an ideal structure for large coercive force and a large magnetic anisotropy. A large magnetic anisotropy is considered to increase resonance frequency of this material. Although a reversible relation of the coercive force was observed for the specimens sintered between 1000 °C and 1200 °C, magnetization results suggest that a sintering temperature of 800 °C is appropriate for the grain growth for the magnetic single domain.

**Figure 4:**
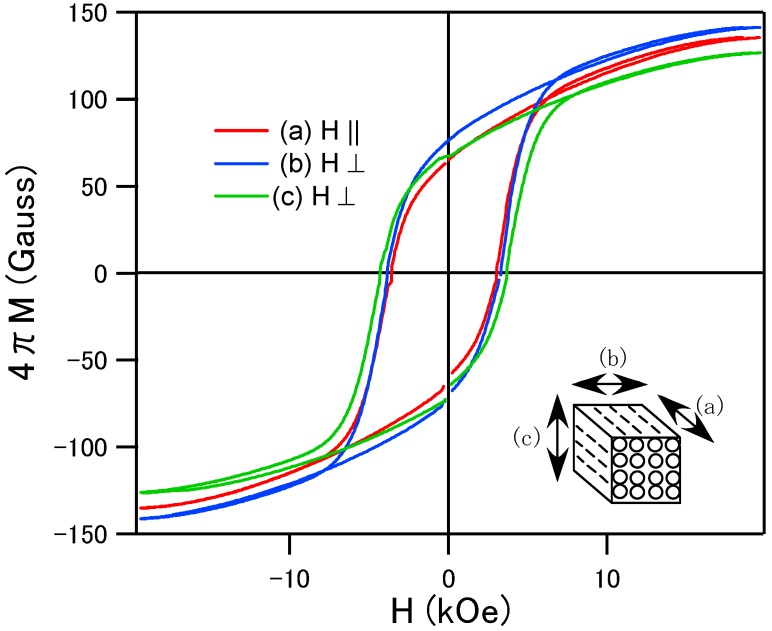
Magnetic hysteresis loops of the porous specimens sintered at 1200 °C in (a), (b) and (c) configurations.

For the characterization of the magnetic anisotropy depending on the direction of the pore, the specimen sintered at 1200 °C (we used this specimen because it’s easier to shape treat than the specimen sintered at 800 °C) was cut into cubes (approximately 5 × 5 × 5 mm) and the magnetic hysteresis curves were measured in the magnetic field parallel and perpendicular to the 1-dimensional pore structure. As shown in Figure 4, the coercive force is approximately 5 kOe and no remarkable differences were observed between parallel (a) and perpendicular (b and c) configurations to the magnetic field. Randomly oriented crystalline structure resulted in the isotropic hysteresis loops. For the case of soft magnetic Ni-Zn ferrite wood, the direction to the porous structure gave the influence for the magnetic anisotropy [7]. The magnetization tends to saturate easily in the magnetic field along the pore structure. We considered that the easy magnetic anisotropy of the porous Ni-Zn ferrite comes from the magnetic shape anisotropy which is generally observed for the case of the thin films. The porous cell wall may behave like a thin film structure. For the case of the Ba-ferrite, the magnetic hysteresis curves were almost isotropic. The hard magnetic properties of this system mainly come from the magnetocrystalline anisotropy. Even if the shape of the cell wall has a roll of film, the effect of the shape magnetic anisotropy can be negligible in comparison with the randomly oriented magnetocrystalline anisotropy. In order to obtain a magnetic easy axis along the 1-dimensional core structures in this system, we have to control c-axis preferred oriented crystallization along the cell wall in the sintering process. At the present stage, characterizations for the high frequency electromagnetic absorber are not enough, however, the porous Ba-ferrite with large magnetic anisotropy can be expected for the absorber in GHz frequency region.

## 4. Conclusions

A porous hexagonal Ba-ferrite was prepared from a cedar wood template by the bio-casting method and its magnetic properties were investigated. Porous structures were maintained in the sintering process and the pore size was approximately 10–20 μm. A large coercive force and anisotropy field was realized at the specimen sintered at 800 °C. Randomly oriented crystalline structure resulted in the isotropic magnetic hysteresis curves. In order to obtain large magnetic anisotropy of Ba-ferrite system by the porous structure, it will be necessary to achieve the c-axis preferential crystallization along the porous structure.
